# Melanotic Neuroectodermal Tumor of Infancy in the Maxilla

**DOI:** 10.1155/2013/726815

**Published:** 2013-09-08

**Authors:** Daniel Falbo Martins de Souza, Daniel Isaac Sendyk, Juliana Seo, Eduardo Vasques da Fonseca, Maria da Graça Naclério-Homem, Maria Cristina Zindel Deboni

**Affiliations:** ^1^Department of Oral Surgery, School of Dentistry, University of São Paulo, Avenida Prof. Lineu Prestes 2227, Butantã, 05508-000 São Paulo, SP, Brazil; ^2^Residency Program in Oral and Maxillofacial Surgery at Conjunto Hospitalar do Mandaqui, São Paulo, SP, Brazil; ^3^Department of Oral Pathology, School of Dentistry, University of São Paulo, São Paulo, SP, Brazil; ^4^Department of Oral and Maxillofacial Surgery, Conjunto Hospitalar do Mandaqui, São Paulo, SP, Brazil

## Abstract

Melanotic neuroectodermal tumors of infancy (MNTIs) are rare fast-growing tumors with high recurrence rates. These tumors, which originate in the neural crest, commonly occur in the anterior maxilla of children under the age of one. Here, we describe an MNTI case in a two-month-old girl with increasing swelling in the left cheek. MNTI was diagnosed in this case following tomography and biopsy. The patient's histological and immunohistochemical profile indicated a remarkable combination of neural, melanocytic, and epithelial cell differentiation. One year following tumor excision, a follow-up examination revealed that the child exhibited no tumor recurrence. Approximately 260 cases of MNTI have been reported since this type of tumor was first described. In the present case, early diagnosis minimized the difficulties and risks associated with treatment and facilitated an optimal outcome. Despite complete surgical excision, careful followup is recommended. In addition, maxillary functional orthopedics and reconstruction may be necessary in cases of MNTI.

## 1. Introduction

Melanotic neuroectodermal tumors of infancy (MNTIs) are rare, fast-growing, melanin-containing lesions that commonly occur in the head and neck regions of children under the age of one [1]. MNTIs are nonulcerative, painless, and pigmented lesions [2], but the pigmentation cannot always be observed through the covering tissues [3]. Uncertainties regarding the histogenesis of MNTIs have led authors in the literature to use a diverse nomenclature, and MNTIs have been described as congenital melanocarcinomas, atypical ameloblastomas and melanocytomas [4, 5]. Despite these controversies, the neural crest is accepted to be the origin of these types of tumors [1, 6–8].

MNTIs generally occur in the maxilla (68%–80%), but they can occasionally arise in the skull (10.8%), mandible (5.8%) or brain (4.3%) [1, 6, 7]. In addition to the head and neck region, other sites can be affected by the condition less frequently, including the femur, epididymis, ovaries, uterus and mediastinum [6, 7]. The effects of gender on MNTI remain controversial, but the majority of publications examining gender differences have reported no significant effects of gender [3].

MNTI lesions are regarded as benign tumors, although they can present locally aggressive behavior, including gradual invasion of the surrounding bone and sinuses. These lesions are characterized by a high recurrence rate that varies between 10% and 60% [6] and the risk of malignant transformation is 6.6% [1, 3, 6]. In plain radiographs, MNTI appears as an intrabony expansive radiolucency, usually with poorly demarcated margins, which likely result from the rapid tumor growth of MNTIs and their tendency to be locally invasive [4, 6, 9–11].

MNTI poses a challenge to clinicians not only in its clinical diagnosis but also in its treatment. Complete surgical excision is generally the first treatment option [12], but protocols combining local surgery and adjuvant chemotherapy have also been proposed for recurrent tumors [13]. The severe adverse effects associated with chemotherapy in young children remain a matter of debate [13–15].

In this report, we present a case of MNTI in a two-month-old baby girl who was treated by complete surgical excision. The clinical, imaging, and histological characteristics of this case are also discussed. Immunohistochemical data regarding MNTI are somewhat inconsistent, so a panel of specific antibodies was performed to correctly identify the different types of tumor cells.

## 2. Case Report

A two-month-old girl was referred to the Oral and Maxillofacial Surgery Department of Conjunto Hospitalar do Mandaqui (São Paulo, Brazil) and presented with a mouth tumefaction with one-month evolution. An extraoral examination revealed facial asymmetry, deletion of the left nasolabial folds, and elevation of the left nasal alar base. Otherwise, the baby appeared to be in a healthy condition and was hydrated.

**Figure 1:**
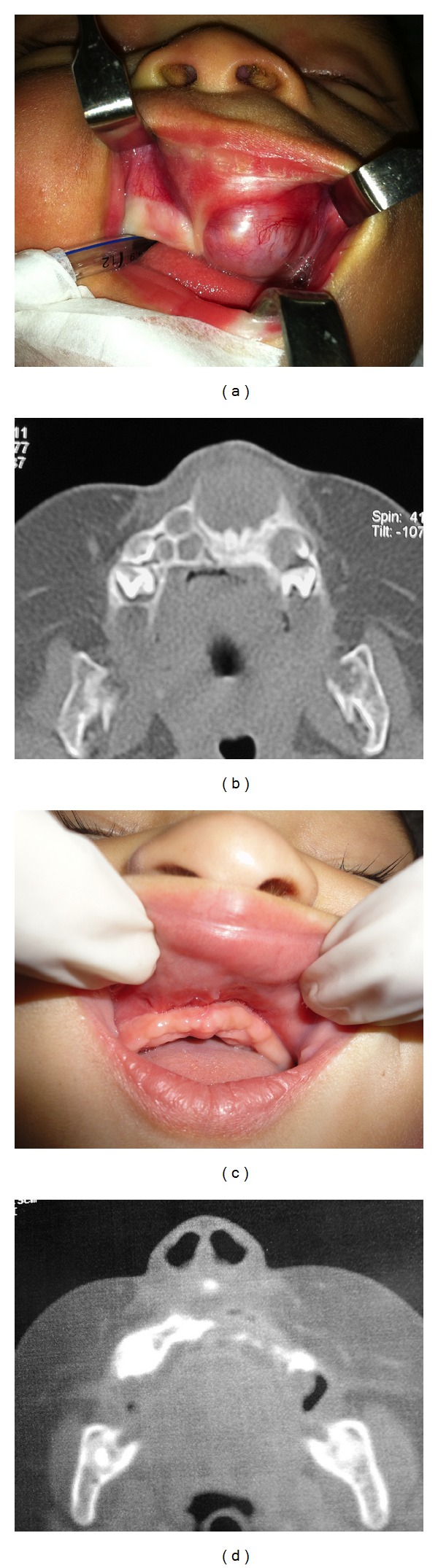
Upon an intraoral assessment, swelling in the left premaxilla alveolar ridge near the canine pillar (a) was observed. Preoperative tomography images (b) revealed a homogeneous hypodense tumor associated with the upper left central primary incisor. An image showing the one-year postoperative intraoral aspect (c). Postoperative tomography image presenting a maxilla defect but no lesion recurrence is shown (d).

During the intraoral assessment, a left premaxilla tumefaction was observed in the alveolar ridge near the canine region (Figure 1(a)). The overlying mucosa was hyperemic, and the labial frenulum was distended. Palpation revealed a lesion with well-defined limits, a smooth surface, and elastic consistency. Tomography images (Figure 1(b)) revealed a homogeneous hypodense tumor that was associated with the upper left central primary incisor. Lesion aspiration produced negative results.

An odontogenic tumor was our first diagnostic hypothesis. A surgical excision was performed under general anesthesia. During surgery, an inner brown-colored aspect of the lesion was observed, which raised the suspicion of MNTI. A peripheral ostectomy was performed to assure total tumor excision.

**Figure 2:**
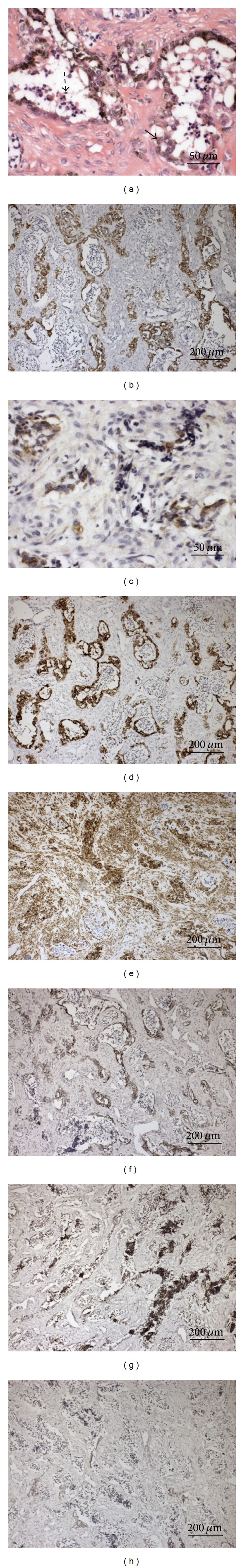
Photomicrographs of histological and immunohistochemical findings. Nests containing biphasic cell populations within dense connective tissue are shown. The presence of small neuroblast-like cells with delicate fibrils between them (indicated by dashed arrows) and large melanin-containing cells (indicated by arrows) were observed (H&E, magnification ×400) (a). Large epithelial-like cells were positive for cytokeratin AE1/AE2 (b) (magnification ×100), epithelial membrane—EMA (c) (magnification ×400), and HMB45 (d) (magnification ×100). Both cell types were positive for vimentin, but the signal intensity was stronger in large cells (e). Both cell types were also positive for chromogranin, but the signal intensity was stronger in small cells (f) (magnification ×100). Synaptophysin was expressed by small neuroblast-like cells (magnification ×100) (g). Nuclear expression of Ki67 was also observed (magnification ×100) (h).

The surgical piece indicated a fibrous blackish-brown lesion containing two primary teeth within the tumor mass. A microscopic assessment revealed fragments of tissue characterized by the proliferation of a dual population of cells arranged in solid nests or cords in the middle of dense, well-cellularized connective tissue (Figure 2(a)). The first cell type consisted of small rounded hyperchromatic cells with minimal cytoplasm that resembled neuroblast-like cells with delicate fibrils between them. The second cell population consisted of epithelioid cells, some of which contained brown intracellular granules, similar to melanocytes. A definitive diagnosis of MNTI was established. To correctly identify the different cell types, an immunohistochemical panel of specific antibodies was performed. Epithelioid cells that contained melanin also expressed cytokeratin AE1/AE3, epithelial membrane antigen (EMA) (Figures 2(b) and 2(c)), strong immunoreactivity for human melanoma black 45 (HMB45) (Figure 2(d)). Vimentin staining was positive for both types of cells, but stronger signal was observed in large cells. Chromogranin expression was also observed in both cell types, but stronger signal was observed in small cells (Figures 2(e) and 2(f)). The small neuroblast-like cells expressed synaptophysin (Figure 2(g)). Tumor cells presented mitotic activity in 4/10 high power fields, and the proliferative activity was approximately 15% as demonstrated by Ki67 staining (Figure 2(h)).

At the time of the one-year follow-up appointment, clinical and tomography examinations did not reveal any tumor recurrence (Figures 1(c) and 1(d)). The child was referred to a maxillary functional orthopedic professional for future attendance and treatment.

## 3. Discussion

MNTI is an unusual type of neoplasm [1, 6–8]. The characteristics observed in the current case report were similar to those that have been described previously in the literature [6–8, 16, 17]. The main clinical, tomographic, and histological aspects were also present.

Although the recurrence rate of MNTI has been reported to be 15% within the first year of enucleation [5, 6], no signs of recurrence were observed after one year of followup in the current case. The 3D topographic reconstruction indicated an anterior maxilla defect but no tumor growth. Most recurrences occur within four weeks of the initial operation [6]. For MNTIs of the jaws, the recurrences are predominantly observed within the first four months following surgery [6, 17].

Although MNTI exhibits some local invasive features, it generally follows a benign course. Several studies [6, 17, 18] have suggested that a conservative surgical approach is usually the best first choice for MNTI treatment. However, the excision with peripheral ostectomy performed in the current case was regarded as a useful treatment option, and it produced complete disease resolution.

Although several authors have reported positive results following surgical treatment and chemotherapy [14, 15], other authors have found radiotherapy and chemotherapy to be ineffective in controlling MNTI [19]. Here, adjuvant chemotherapy was not advocated, and we believe that it should only be considered when an aggressive recurrence or malignant transformation occurs. Serious adverse effects are generally associated with chemotherapy in young children [13, 18]. Therefore, chemotherapy should be avoided to prevent these adverse effects, and surgical excisions with peripheral ostectomy are curative in most cases [6, 11].

MNTIs are biphasic tumors composed of small-cell and large-cell components that are arranged in nest or cord arrangements set in a vascularized fibrous stroma [17]. The nests of small cells are usually surrounded by large cells that form structures resembling gland tissue [11]. The large, cigar-shaped, elongated melanin granules that are commonly observed in MNTI differ from those observed in melanoma, in which the granules are smaller or are unpredictably sized inclusions in melanophages [20].

Immunohistochemical staining in MNTI is somewhat variable and can be used to assist diagnoses [1–3, 8]. Prior studies have associated MNTI with retinal anlage tumors [3]. Cytokeratins 7, 8, 18, and 19, which are all expressed in MNTI, are also expressed by cultured retinal pigment epithelial cells [3].

In the present case the expression of synaptophysin was more frequently detected in small cells than in larger cells. Chromogranin, another neuroendocrine marker expressed by both cell types, was expressed at a greater intensity in the small neuroblast-like cells. Earlier chromogranin assays were negative in many MNTI studies [21, 22].

Large epithelioid cells typically express cytokeratin, HMB45, and EMA but are rarely positive for S100 protein and are usually negative for melan-A, glial fibrillary acidic protein (GFAP), and alpha-fetoprotein (AFP). S100 is expressed by melanocytes and melanomas. The immunohistochemical profile of MNTI is generally positive for cytokeratin and HMB45 and negative for S100 [3, 8]. In the present report, the immunohistochemical patterns observed were somewhat similar to published reports and validated the neural origin of the tumor.

The expressions of Ki-67 and CD99 are quite uncommon and might be related to more aggressive tumor growth [1, 6]. Ki-67 is a nonhistone nuclear antigen and a component of the protein structure known as the chromosome support. Ki-67 expression is determined by a gene located in 10q25 and occurs in the final step of G1, S, G2, and M. Thus, Ki-67 expression is often used to assess the proliferative fraction of potentially aggressive tumors [8]. In addition, the CD99 protein is involved in T lymphocyte signaling, and CD99 positive staining has been linked to aggressive MNTI [3, 19].

Ki-67 expression was observed to be elevated in 15% of the histological fields observed in this report, a finding that indicates a slight potential for a more aggressive lesion. Fortunately, the treatment approach employed here, which included a marginal ostectomy, was adequate even despite the high rate of mitosis.

Although our data reveal that the risk of recurrence is significantly reduced after one year, a rigorous followup should be performed over a longer period of the child's development. The prospect of local reconstruction has been planned, and maxillary functional orthopedic treatment will likely be required to stimulate adequate facial growth as fibrous healing is a considerable local issue.

In view of the rapid growth of MNTIs, their malignant potential, and their high rate of recurrence, it is essential to diagnose this type of tumor at an early stage. Pediatricians and pediatric dentists should be aware of this disease and direct patients to prompt oral surgery treatment to minimize mutilating surgeries.
